# Eplerenone Treatment in Chronic Central Serous Chorioretinopathy

**DOI:** 10.7759/cureus.18415

**Published:** 2021-10-01

**Authors:** Faisal Iqbal, Kashif Iqbal, Bilal Inayat, Sabeen Arjumand, Zarish Ghafoor, Werdah Sattar, Kiran Abbas

**Affiliations:** 1 Department of Ophthalmology, Layton Rahmatulla Benevolent Trust (LRBT) Eye Hospital, Lahore, PAK; 2 Department of Pharmacology, Sharif Medical and Dental College, Lahore, PAK; 3 Department of Pharmacology, Avicenna Medical College, Lahore, PAK; 4 Department of Ophthalmology, Lahore General Hospital, Lahore, PAK; 5 Department of Medicine, Jinnah Postgraduate Medical Centre, Karachi, PAK

**Keywords:** srf, cscr, macula, choroidal thickness, eplerenone

## Abstract

Background

The study examined the efficacy of eplerenone in the management of chronic central serous chorioretinopathy (CSCR) with the aim of short-term observations. The study also aimed at observing changes in optical coherence tomography (OCT) parameters and visual acuity.

Methodology

This retrospective study was conducted at Layton Rahmatulla Benevolent Trust (LRBT) Eye Hospital from September 2019 to October 2020. A thorough ocular examination, color fundus photographs, fluorescein angiography, and macular OCT were performed on all patients. We administered one tablet of 50 mg eplerenone on day one and further advised the use of the same dose for 30 days. After the administration of the tablet, the patients were further analyzed on weeks one, two, and four. On every visit, we examined ophthalmic conditions by visual acuity, slit lamp, and dilated fundus examinations along with macular OCT and measured blood pressure. At follow-up, we measured the levels of serum creatinine at weeks one and four. Student's t-test and chi-square test were used for normal distribution and nominal variables. A p-value < 0.05 was considered statistically significant in all the analyses.

Results

A total of 15 patients were selected for this research, but unfortunately, two of them withdrew amid the study. For the remaining 13 patients, the mean duration of observing symptoms was three months and three weeks. At one-month follow-up, the mean subretinal fluid (SRF) height (94.18 μm) decreased, but we did not find any statistical significance between the SRF height at one-month follow-up and baseline (113.15 μm). In four patients, the SRF height increased up to 3-30 μm after four months of treatment. In our study, we found some negative consequences of eplerenone therapy in terms of hypertension, cramps, nausea, and migraine.

Conclusion

We concluded that short-term eplerenone treatment assists in the reduction of the choroidal thickness (CT) and central macular thickness (CMT) among patients with central serous chorioretinopathy. However, eplerenone treatment failed to decrease subretinal fluid height and does not bring any significant improvement in the visual acuity of patients. Some mild adverse effects of the treatment include hypertension, abdominal cramps, nausea, and migraine.

## Introduction

One in every 1000 individuals suffers from central serous chorioretinopathy (CSCR) per year [1]. CSCR is a condition of the retina and choroid in which an increased amount of serous fluid gathers under the macula [2]. Most Asian and Caucasian populations are prone to this disease [3]. Abnormalities in choroidal vascular permeability and dysfunction of retinal pigmented epithelium (RPE) pumps are the major causes of CSCR. This triggers the growth of serous fluid under the retina, which is the leading clinical feature of CSCR [3,4].

Optical coherence tomography (OCT) is considered one of the best tools for diagnosing CSCR. It also assists in the evaluation of disease activity. In some research, OCT helps in detecting the thickness of the retina and subretinal fluid (SRF) at different time intervals [5]. In acute cases, a significant decline in SRF height was observed within six to 12 weeks [3]. In some cases, the reabsorption of SRF takes place between 12 and 24 weeks [6]. Chronic CSCR has not yet been properly defined, but the majority of physicians define it as a condition in which the SRF lasts more than three months and can reoccur within one year [3,4]. The majority of patients with CSCR are asymptomatic, raising the probability of the recurrence of the disease by 30%-50% [7,8]. This recurrence can be observed in the same eye, opposite eye, and even in both eyes [3,4].

Another major cause of CSCR is increased mineralocorticoid activity, prompting the investigation of inhibitors as a potential therapy for CSCR [9,10]. Many ophthalmologists use eplerenone as a treatment for CSCR due to its fewer side effects [9-11]. Many double-blinded placebo studies that gave contradicting results of eplerenone at different time intervals were conducted in the past [12-14]. Therefore, we conducted this research to examine the efficacy of eplerenone in the management of CSCR with an aim of short-term observations. It was our objective to administer eplerenone therapy for four weeks and observe the changes in OCT parameters and visual acuity.

## Materials and methods

This retrospective study was conducted at Layton Rahmatulla Benevolent Trust (LRBT) Eye Hospital from September 2019 to October 2020. We selected patients from the young to the old age group to achieve good results. Patients above 18 years old and suffering from chronic central serous chorioretinopathy (CSCR) were included. CSCR was diagnosed on the basis of the characteristic clinical and imaging findings. We defined chronic CSCR as a condition in which patients were suffering from constant SRF for more than three months confirmed by OCT after the emergence of CSCR symptoms. We have included only one CSCR eye of each selected patient, and in the case of CSCR in both eyes, only the clinically worse eye of the patient was included in this research. All pregnant women, patients who suffered from concomitant eye diseases, and patients who already have the worst condition of the macula were excluded from this research. We further assured that all patients with type 2 diabetes and those with ≥5.0 mEq/L potassium, >2 mg/dL creatinine, and <50 mL/min creatinine clearance at baseline were excluded in this research. All patients who were taking potassium supplements and CYP3A4 inhibitors were also excluded.

Written consent was acquired from all participants of the study after explaining the purpose of our study. Information related to their family history of the disease, previous treatment for CSCR, and current medications were also noted. 

The Early Treatment Diabetic Retinopathy Study (ETDRS) chart was used to detect the best-corrected visual acuity (BCVA). We further performed high-resolution OCT scans to measure the maximum height of the subretinal fluid (SRF). Baseline characteristics were further observed, along with the side effects of therapy.

Before initiating our treatment, we continuously observed blood pressure for one week. All patients underwent a thorough ocular examination, color fundus photographs, fluorescein angiography, and macular OCT. We administered one tablet of 50 mg eplerenone on day one and further advised the use of the same dose for 30 days. After the administration of the tablet, the patients were further analyzed at weeks one, two, and four. We examined ophthalmic conditions and measured blood pressure on every visit. These conditions included visual acuity, slit lamp, and dilated fundus examination, along with macular OCT. During the follow-up, we measured the levels of serum creatinine at weeks one and four [15].

All the collected information was analyzed using the Statistical Package for the Social Sciences (SPSS) version 23.0. Nominal variables were presented as percentages, whereas continuous variables were presented as means and standard deviations. Non-normally distributed variables were analyzed using the Mann-Whitney U test, whereas Student's t-test and chi-square were used for normally distributed and nominal variables. A p-value < 0.05 was considered significant in all the analyses.

## Results

A total of 15 patients were selected for this research, but unfortunately, two of them withdrew during the study. For the remaining 13 patients, the mean duration of symptoms was observed to be three months and three weeks. All our selected participants were male, and five of them already received central serous chorioretinopathy (CSCR) treatment in the past. Three of them took intravitreal bevacizumab as a treatment for one month, 11 months, and 34 months, respectively, before eplerenone therapy. The remaining two patients received nine months of treatment of focal laser therapy. Thirteen patients completed a four-week course of the treatment. Two patients continued this treatment for seven and 20 weeks, respectively, as per their own choice (Table 1).

**Table 1:** Demographic Characteristics of Patients (n = 13) CSCR: central serous chorioretinopathy

Variables	Total number	Mean and standard deviations
Sex		
Male	13 (100%)	
Female	0	
Mean age (years)	-	55.61 ± 2.3 (range: 45–71)
Intravitreal bevacizumab	3 (23.07%)	
Total number of patients with prior treatments	5 (38.46%)	
Focal laser therapy	2 (15.38%)	
Duration of CSCR symptoms prior to eplerenone therapy (months)		17.40 ± 3.9 (range: 4–36)

During the one-month follow-up, the mean subretinal fluid (SRF) height (94.18 μm) decreased, but unfortunately, we did not find any statistical significance between the SRF height at one-month follow-up and baseline (113.15 μm). In four patients, the SRF height increased up to 3-30 μm after four months of treatment. All these patients already underwent various treatments. After four weeks, choroidal thickness (CT) and the mean central macular thickness (CMT) decreased in comparison with baseline after four months of treatment. We observed central macular thickness and performed enhanced depth imaging optical coherence tomography (EDI-OCT) scans before and after treatment. Two patients who continued treatment for more than four weeks experienced an increase in SRF height, whereas the best-corrected visual acuity (BCVA) was unchanged in both patients before treatment. In our study, we found some negative consequences of eplerenone therapy in terms of hypertension, cramps, nausea, and migraine (Table 2).

**Table 2:** Optical Coherence Tomography (OCT) Values, Visual Acuity, and Laboratory Values of Selected Participants *Statistically significant

Variables	Four weeks after treatment	Baseline	p-value
Central macular thickness (μm)	339.46 ± 27.29	365.23 ± 26.83	0.04*
Nasal choroidal thickness (μm)	394.89 ± 17.22	410.00 ± 20.36	0.14
Subretinal fluid height (μm)	94.18 ± 17.53	113.15 ± 18.69	0.08
Temporal choroidal thickness (μm)	395.96 ± 15.69	411.07 ± 21.17	0.33
Visual acuity, LogMAR (Snellen equivalent)	0.18 ± 0.08 (20/30)	0.15 ± 0.08 (20/28)	0.16
Subfoveal choroidal thickness (μm)	422.20 ± 18.23	452.07 ± 19.70	0.002*
Diastolic blood pressure (mmHg)	83.00 ± 2.91	86.61 ± 2.71	0.31
Serum potassium (mEq/L)	4.38 ± 0.07	4.27 ± 0.11	0.11
Systolic blood pressure (mmHg)	127.69 ± 4.55	134.76 ± 5.44	0.10
Serum creatinine (mg/dL)	0.97 ± 0.06	0.92 ± 0.04	0.82

## Discussion

The primary concern of this study was to observe the changes in the subretinal fluid after receiving one month of treatment of oral eplerenone. The results of our study depict a decline in the subretinal fluid (SRF) height (16.77%) after one month of treatment (94.18 ± 17.53 μm). On the other hand, we did not observe any significant change from the baseline characteristics of the patients after treatment (113.15 ± 18.69 μm). Our results, unfortunately, were not statistically significant. These results are quite unrelated to the previous study of Bousquet et al., in which no significant change in SRF height was observed after one month [16]. Our results, however, are in accordance with some previous studies, in which a relevant inclination in SRF height was observed after one month of taking oral eplerenone treatment [9,12,17]. In a study by Rahimy et al., after two months of treatment, a 62.81% reduction in SRF height was observed [13], whereas, in the placebo study of Schwartz et al., a decrease in SRF height after one week of treatment was observed [12]. Both studies did not observe any significant change in baseline compositions.

Our results observed a significant decrease in central macular thickness (CMT) (7.05%) and subfoveal choroidal thickness (CT) (6.6%) after one month of treatment [12,13,16-19]. These results are in accordance with some previous studies. In the study of Bousquet et al., a 30.11% decrease in CMT was observed during one month of treatment [16]. He further observed this decrease for three months, but unfortunately, he did not find any significant difference in the CMT of the eplerenone group and the placebo group [12]. A decrease in subfoveal CT was also observed in many previous studies [9]. The results obtained from the study of Ghadiali et al. were completely different compared with the results of our study [20]. They reported an increase in SRF height but could not find any changes in CMT and CT in 12 months of treatment of mineralocorticoid antagonists among the patients of chronic central serous chorioretinopathy. The study by Swartz revealed nonsignificant changes in CT and found a decline in SRF height and CMT in patients treated with eplerenone [12].

We did not find any significant change in visual acuity after one month of treatment. This happened due to the 20/20 vision of seven out of 13 patients (53.8%) before treatment of our selected group. On the other hand, only six patients had worse than 20/20 vision, and improvement occurred only in four patients after treatment. Therefore, we did not find any significant correlation between visual acuity, SRF height, and CMT. We observed that visual acuity may be reported by the patients after several months due to fluid resorption. This depicts that visual acuity is not a good marker of visual impairment in these patients [21]. In patients with CSCR, the retinal pigmented epithelium is significantly related to the visual impairment of patients treated with eplerenone [18]. One of the results of the present study is in correspondence with the previous research of Schwartz et al. and Bousquet et al., in which they did not report significant BCVA improvement among patients with chronic CSCR even after 30 days of spironolactone treatment [12,22]. The results of their studies were caused by a limited sample size and high BCVA baselines. A meta-analysis on mineralocorticoid receptor antagonists reported remarkable improvement in BCVA within one to two months of treatment [14]. However, in the study of Rahimy et al., a high ratio of visual acuity improvement was reported (64%) within nine weeks after eplerenone treatment [13]. On the other hand, the study of Daruich et al. and Zola et al. found improvement in visual acuity in six- and 24-month follow-up [9,23]. All these results depict that long-term treatment may improve BCVA outcomes. 

In our study, four patients (30.76%) were identified as nonresponders. Nonresponders were defined as patients in which no changes were observed during treatment but more SRF was observed after treatment. In these four patients, we observed increased SRF height after treatment as compared with baseline. In one patient, treatment was administered for seven weeks, but still, we observed a constant increase in SRF height. Our results are parallel to those studies that observed 30.8% and 33.3% nonresponders to CSCR treatment [12,24]. This happened due to the longer duration of the disease. A study by Rajesh et al. observed that the baseline characteristics are the best predicting factors for describing eplerenone efficiency [25]. In our study, we found some negative consequences of eplerenone therapy in terms of hypertension, cramps, nausea, and migraine, which are also reported in some previous studies.

## Conclusions

We concluded that short-term eplerenone treatment assists in the reduction of choroidal thickness and central macular thickness in patients with central serous chorioretinopathy. However, eplerenone treatment failed to decrease subretinal fluid height and does not bring any significant improvement in the visual acuity of patients. Some mild adverse effects of the treatment include hypertension, abdominal cramps, nausea, and migraine.
